# Effectiveness of an Interactive Mobile Health Intervention (IMHI) to enhance the adoption of modern contraceptive methods during the early postpartum period among women in Northeast Ethiopia: A cluster Randomized Controlled Trial (RCT)

**DOI:** 10.1371/journal.pone.0310124

**Published:** 2024-11-14

**Authors:** Niguss Cherie, Muluemebet Abera Wordofa, Gurmesa Tura Debelew

**Affiliations:** 1 Population and Family Health Department, Faculty of Public Health, Institute of Health, Jimma University, Jimma, Ethiopia; 2 Reproductive and Family Health Department, School of Public Health, College of Medicine and Health Sciences, Wollo University, Dessie, Ethiopia; Wollega University, ETHIOPIA

## Abstract

**Background:**

Women in the early postpartum period face substantial unmet needs in contraception to encourage birth intervals and reduce unintended pregnancies. The widespread ownership of mobile devices offers an opportunity to employ mobile health strategies for enhancing communication between healthcare providers and clients. However, little is known about the effectiveness of mobile health interventions to improve early adoption of contraceptive methods after childbirth in Ehiopia.

**Objective:**

This study aimed to evaluate the effectiveness of a mobile health intervention in enhancing the uptake of modern contraceptive methods in the early postpartum period in Dessie and Kombolcha cities, northeast Ethiopia.

**Methods:**

The research was conducted in Dessie and Kombolcha cities zones located in the Amhara region of Northeast Ethiopia from 15^th^ January to 15^th^ June, 2023. Pregnant women with a confirmed gestation of 30 weeks were enrolled and followed up to the 45-day postpartum period. The study employed a cluster randomized control trial involving 764 participants (381 controls and 383 in the intervention group). The intervention group received a new mobile health intervention in addition to the existing healthcare practices, while the control group solely adhered to the current healthcare practices. Data were collected using the Open Data Kit (ODK) and exported to STATA 17 for analysis. The marginal model Generalized Estimating Equations (GEE) through the application of an exchangeable working correlation was applied. The effect of the intervention on the outcome was measured using the odds ratio with a 95% confidence interval at a p-value less than 0.05 significant level.

**Results:**

The study found that 78.7% of participants in the control group and 77.3% in the intervention group had sexual practice after childbirth. The proportion of early postpartum contraceptive uptake in the intervention group (51.6%) was significantly higher than in the control group (38%). The odds of adopting modern contraceptive methods during the early postpartum period were 1.6 times higher among mothers who received the mHealth intervention compared to those in the control group (AOR: 1.6, 95% CI: 1.249–2.123). The study identified significant predictors for the uptake of contraceptive methods during the early postpartum period, including having a live newborn (AOR: 3.7, 95% CI: 1.034–13.353), parity (AOR: 1.7, 95% CI: 1.069–2.695), and previous experience with contraceptive initiation (AOR: 0.5, 95% CI: 0.358–0.912).

**Conclusion:**

This study findings demonstrated that the potential effectiveness of mobile health interventions in promoting timely contraceptive adoption during early postpartum period. The mobile health intervention, combined with factors such as timing of previous contraceptive initiation, newborn status, and maternal parity, significantly enhances the likelihood of early contraceptive adoption. These nuanced insights provide a strong foundation for developing targeted health interventions and policies aimed at improving early postpartum contraception.

**Registration:**

The trial was registered on December 23, 2022, in the Protocol Registration and Results System (PRS) Clinical Trial Registry, www.ClinicalTrials.gov, ID: ClinicalTrials.gov ID: NCT05666037.

## Introduction

Early postpartum contraceptive adoption is defined as women who have ever used any kind of modern birth control method within the first six weeks after they gave birth [1, 2]. Early Postpartum family planning contributes to the reduction of narrow birth intervals, and unplanned and unintended pregnancy further contributes to the reduction of maternal and newborn deaths. Postpartum women have among the highest unmet needs for family planning to promote longer birth intervals [3].

Evidence shows that short birth intervals increase the danger of maternal, newborn, neonatal, and associated under-5 mortality [4] and is related to the magnified risk of preterm birth, low birth weight, stunting, and skinny youngsters [5–7]. To reduce the danger of adverse maternal, perinatal, and neonatal outcomes, the World Health Organization (WHO) recommends a minimum of 24–36 months interval between delivery and the later gestation [8, 9]. Early postpartum contraception is a well-tried and cost-efficient intervention to stop each maternal and newborn death by reducing the short birth intervals, the number of abortions, and the proportion of births at high risk [10].

The early post-partum period provides a unique opportunity to meet the reproductive health needs of women particularly the need for contraception after childbirth. The timing of the return of fertility after childbirth is variable and unpredictable. Some women resume ovulation and menstruation as early as 28 days post-delivery [11]. Consequently, gaps exist in meeting the demand for contraception among women of reproductive age, particularly in the early post-partum period [12].

The promotion of early postpartum contraception in countries with high birth rates has the potential to avert 32% of all maternal deaths and nearly 10% of childhood deaths [13]. Early postpartum Contraceptive prevalence is still relatively low (38.5%) in Ethiopia, and the unmet need (25%) [14]. Nearly half (47%) of postpartum women have short (<23 months) birth-to-pregnancy intervals in Ethiopia [15].

The Ethiopian government is putting great efforts into programmatic and policy initiatives in place to increase access to and utilization of early postpartum contraceptive methods through the rapid expansion of primary health care facilities, massive training of midwives, and free provision of family planning services [16]. However, the level of early postpartum contraceptive method uptake is still unacceptably low in comparison with the sustainable development target. Despite the national efforts Ethiopia continues to have an unmet contraceptive need and a high rate of maternal morbidity and mortality associated with pregnancy, childbirth, and postpartum [17].

Women received guidance and counseling concerning narrow birth prevention throughout the antepartum and immediate postnatal period [18]. However, once discharged, most women don’t come to health facilities for follow-up visits to birth prevention service providers [19]. Because of the high proportion of postpartum women lost to follow-up at health facilities the potential for postnatal narrow birth prevention has not been realized [20]. The growth and access for mobile phones and mobile services and unexampled increase in mobile penetration are anticipated to facilitate the use of mHealth initiatives in resource-restricted settings [21, 22]. Extending the reach of the healthcare system, mHealth is intended to function as a cue to action and boost communications to support healthcare behavior amendment [23]. High mobile phone ownership presents a chance to utilize mHealth approaches to push behavior amendment and reminder intervention on maternal and child health care in the community [24, 25].

There is growing evidence showing that ordinarily utilized mobile health solutions (mHealth) like Sending Message Service(SMS) are used to improve health service delivery processes and health outcomes within the developed world [26]. However, no evidence demonstrates the effectiveness of mobile health interventions on key maternal and child health service outcomes in Ethiopia.

This study aimed to incorporate the family planning counseling guideline with the mobile short message with the hypothesis that such intervention would be effective in enhancing the uptake of contraceptive methods during the early postpartum period. Thus, the target of the planned study was to evaluate the effectiveness of mobile health intervention to enhance early postpartum modern contraceptive method adoption among mothers in Dessie and Kombolcha city zones, Northeast Ethiopia. The findings of this study would be expected to contribute to the existing knowledge gap, to understand the possible technology-based interventions for behavior change in the community, and to act accordingly. Additionally, the findings will be used as baseline information for improving healthcare services to policymakers, reproductive health programmers, program implementers, NGOs, local health planners, and healthcare providers.

## Methods and materials

### Study area, design, and period

The study was conducted in the Dessie and Kombolcha city zones in Amhara regional state, Northeast Ethiopia. Dessie is the administrative town of the south Wollo zone, which is situated 401 KM from Addis Ababa to the north. Dessie city is split into 5 sub cities with 22 kebeles and has 2 governmental hospitals and 8 health centers. Based on population projection for 2023 more than 470,000 residents population with an estimated 21, 620 pregnant women in Dessie town. Kombolcha town is 30 km from Dessie city and 375 km from Addis Ababa is an industrial zone and dry port in northeast Ethiopia. There are more than 350,000 resident populations with 16,100 estimated pregnant women in Kombolcha town. It is divided into 5 sub cities with 22 kebeles and has one governmental hospital and five health centers [27]. Cluster randomized control trial study was conducted from 15^th^ January to 15^th^ June, 2023.

### Population and eligibility

**Fig 1:**
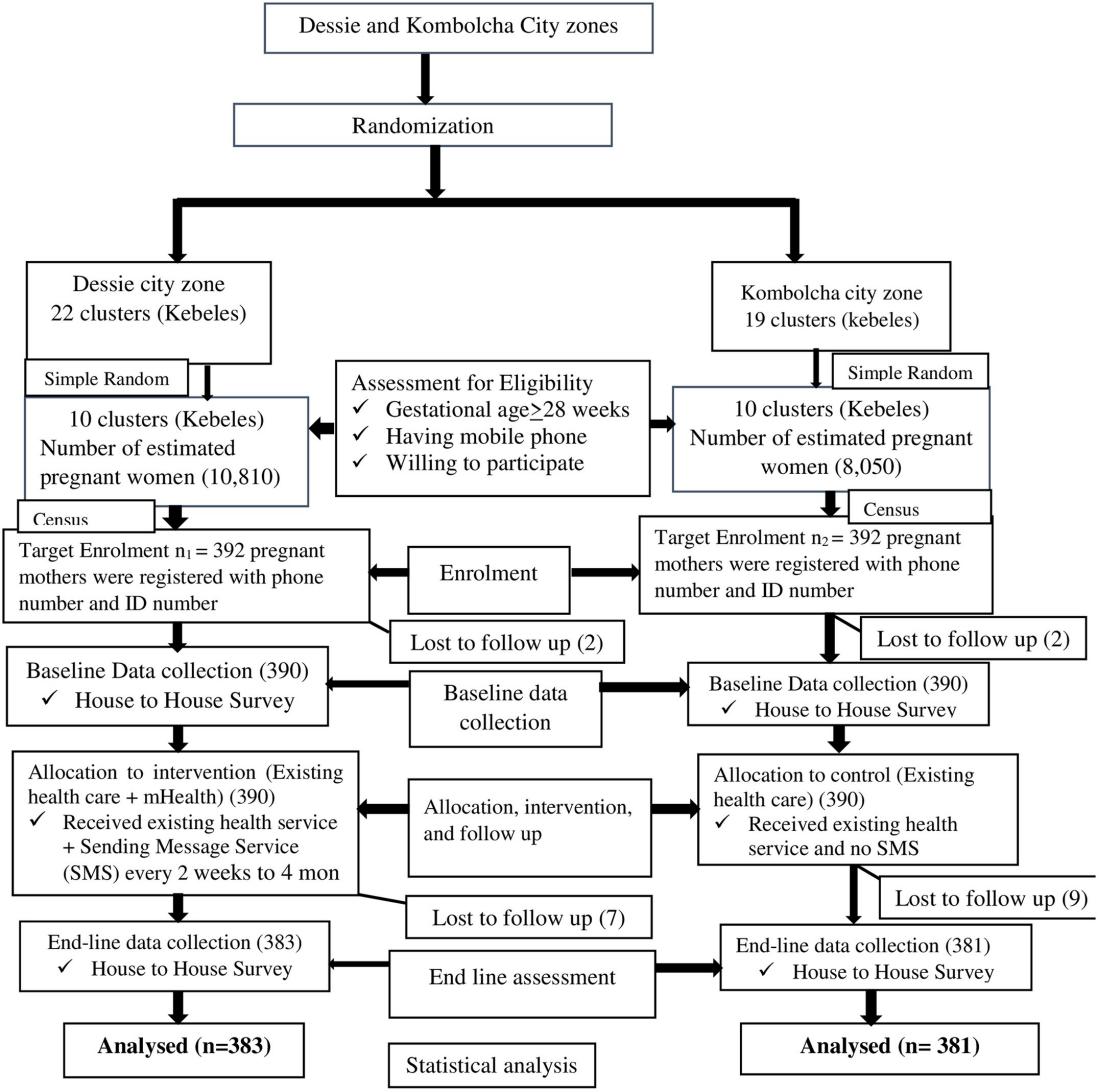
Consolidated Standards of Reporting Trials (CONSORT) diagram depicts the sampling method and allocation of study units, assessment of eligibility criteria to include study participants to the effectiveness of mHealth intervention to improve adoption of the contraceptive method during the early postpartum period in Dessie and Kombolcha city zones, Northeast Ethiopia.

First census was conducted to identify eligible pregnant women and a baseline study was done. All eligible pregnant women based on world health organization pregnancy screening eligibility criteria at 30 weeks gestational age were included in the intervention and control group then followed up to 6 weeks postpartum. All post-partum women who were in the follow up were included in the end line study (Fig 1).

### Sample size determination and sampling procedures

The sample for the study was determined using the assumption of superiority trial design using STATA 17 to demonstrate the superiority of a new intervention compared to the existing early postpartum contraceptive adoption increased from 38.5% to 48.5% [14]. The following assumptions were used to calculate the sample size. Error probabilities (0.05), Power (80%), the ratio of clusters (1), Effect size proportion to control (38.5%), Effect size proportion to experiment (48.5%), and intra-class correlation (10%). The total sample size with a 10% non-response rate of study subjects was 784(392 interventions and 392 controls). A cluster sampling technique was applied. First, clusters (Kebeles) were randomly selected and the census was conducted to identify and register pregnant women based on eligibility criteria. All registered eligible pregnant women in the selected clusters (Kebeles) were included in the baseline study.

### Randomization to intervention or control group assignment

There is a 30 km buffer zone between Dessie and Kombolcha towns to prevent information contamination of the intervention. After baseline data collection intervention and control groups were allocated through stratified randomization of clusters. The kebeles in Dessie and Kombolcha cities were identified as clusters. Clusters were stratified based on the average number of pregnant women served per month and geographic location. Within each stratum, randomly assign clusters to either the intervention group or the control group using a computer-generated randomization sequence to ensure the randomization process was unbiased to minimize confounding factors and ensure a balanced distribution of health facilities across intervention and control groups, enhancing the reliability and validity of the study results. Outcome assessors were blinded to the allocation to prevent assessment bias.

### Recruitment and participant timeline

Pregnant women with 26–28 weeks of gestation (based on WHO eligibility criteria) were recruited and baseline data was collected from selected clusters. Women who were provided informed consent were asked to complete a post-consent eligibility assessment including access to a mobile phone, willingness to participate in the follow-up study and to receive health messages on their mobile phone. Women who meet these eligibility criteria were enrolled in the study and administered the baseline assessment. The intervention was started among pregnant women at 30 weeks of gestation and continued up to 6 weeks of the postpartum period for 4 months. The result of the outcome was measured two times. First the base line assessment was conducted prior to the intervention’s commencement, capturing participant characteristics from 1^st^ -15^th^ January, 2023. Intervention was done from 15^th^ January to 15^th^ May, 2023. Second end line data collection point typically occurred at the end of the study period from 25^th^ May to 15^th^ June, 2023.

### Description of the intervention

The mobile health intervention for enhancing the early adoption of modern contraceptive methods among postpartum mothers was a behavior change and reminder initiative designed to enhance maternal and child health outcomes. The intervention was developed using the Trans theoretical Model (TTM), which is also known as the theory of Change model. This model allows for personalized strategies that address readiness and barriers to change, making it suitable for designing effective interventions in health behavior, including contraception adoption through mobile health platforms.The intervention group received a series of text messages (SMS) promoting behavioral change and encouraging the uptake of modern contraceptive methods during the early postpartum period. In contrast, the control group relied solely on routine healthcare providers at health facilities without any mobile-based interventions. During the study, participants in the intervention group received one text message every two weeks up to 42 days following delivery. Trained 3 female health workers, proficient in the local language, delivered the intervention. Each participant received a total of eight SMS over the four months. In cases where women possessed mobile phones but lacked formal education or the ability to read the messages, they were connected with their husbands or nearest family members, who would read the messages to them.

Researchers developed early postpartum family planning intervention mobile health messages from culturally congruent family planning behavior change framework, and national and WHO family planning guidelines [27, 28]. The messages consist of a congratulatory message, counseling on maternal and neonatal health needs, the dangers of narrow birth intervals, time of fertility return after childbirth, and planning future pregnancies. All messages were developed in English and later translated into the local language Amharic. Additionally, researchers gathered feedback from experts and used findings to further refine the behavior change intervention messages. The appropriate mobile health intervention messages provided at each schedules are described (Supporting information 1). The strategies used to maintain intervention fidelity were comprehensive training sessions for individuals who delivered the intervention, training manuals, and protocols, developed scripts, timing guidelines, and instructions for handling common issues that may arise during delivery. Conduct regular supervision and monitoring visits to observe the intervention delivery and provide feedback.

The investigators employed various assessment techniques to determine whether the intended content reached the audience. The mobile health (mHealth) platform used in the study have been included mechanisms to confirm message delivery. This could involve tracking delivery status (e.g., sent, delivered) of intervention messages to participants’ mobile phones. Follow-up surveys have been conducted to gather participant feedback on their interaction with and understanding of the intervention messages at the end line evaluation.Self-report measures from implementers were used to capture their adherence to the intervention procedures and any challenges faced.

### Operational definitions

#### Early postpartum contraceptive method adoption

Early postpartum contraception is outlined as women who have ever used any kind of modern birth control technique at intervals during the first six weeks when she gave birth [29]. If the respondent answers yes it is coded as "1" and if not coded as "0".

#### Mobile health (mHealth)

Mobile health (mHealth) refers to the employment of wireless, moveable data and Communication Technologies (ICT) to support health and health care. For this study, mobile health includes sending message service (SMS) on early post-partum modern contraceptive method adoption for behavior change intervention and reminded [26, 30, 31].

#### Women autonomy

We use 23 items applied considering the three categories namely decision-making autonomy, movement autonomy, and financial autonomy. Principal component analysis method with a fixed number of factors for measuring women’s autonomy in the context of developing countries. Those will have mean/median and above values will be taken as autonomous [32].

#### Wealth index

We use 19 items applied considering the urban wealth assessment tool. Principal component analysis method with a fixed number of factors for measuring wealth index in the context of developing countries. Then categorized as rich, middle, and poor based on the percentile value of the score [33, 34].

### Data collection tools and procedures

Data were collected by using interviewer-administered structured questionnaires adapted from different kinds of literature [2, 14, 29, 30, 35–37]. A form on the Open Data Kit (ODK) was created, and data collection utilized the ODK Collect tool, with the aggregated data subsequently compiled on the KOBO Toolbox URL: https://kc.humanitarianresponse.info/nigucheru2015. Participant recruitment and baseline data collection were done by eight trained nurses. End line survey was conducted by eight female nurses well familiar with local geography and who were not involved in recruitment, baseline data collection, and intervention process. Before actual data collection, the census was conducted and a list of eligible pregnant women with important contact and follow-up addresses was obtained from selected clusters. Then, a specific identification number (code) was given to all the registered pregnant women to avoid identifiers and to link the data during the intervention and follow-up. Following this actual baseline data were collected from home to home in the community and endline data were collected after 45 days of childbirth.

### Data quality assurance

Data collectors received training and participated in pretests. All the questionnaires were prepared in English, then translated into the local language Amharic, and translated to English to check their consistency. Validating and ensuring reliability of outcome measures in Amharic language was applied through translation and cultural adaptation processes, followed by rigorous testing and validation among the target population to ensure accuracy and consistency of measurement during feasibility pretest study.

Intra-variability of interviewers was assessed by comparing data from supervisors and data collectors. Four Master of Public Health (MPH) holders, alongside the principal investigator, supervised the entire data collection process. Any inconsistencies were addressed promptly. Throughout the intervention and study period, the principal investigator and supervisors ensured adherence to protocols and closely monitored data collection. The research team tracked successful message delivery, intervention completion, and participant dropouts. The tool was validated before and during baseline assessment. The validity of measures used in the study was established through content and construct validity. Internal consistency reliability was assessed using statistical measure Cronbach’s alpha which was 0.82 for scales or measures with multiple items.

### Data processing and analysis

Data from ODK were exported to STATA 17. Descriptive and summary statistics were done. In clustered data, observations are usually taken from the same unit, and thus this information forms a cluster of correlated observations. Sub-group analysis was done based on baseline characteristics, such as age, socio-economic status, educational level, geographic location, parity, or initial contraceptive use. Stratified analysis by subgroup variables and interaction terms in regression models was done to test for significant differences in intervention effects between subgroups. The marginal models Generalized Estimating Equations (GEE) were done by using STATA 17. This model was preferred to avoid the clustering effects as the factors exist at different levels and violate the assumption of independence for the ordinary logistic regression. Although the participants were randomly allocated to study groups, estimates were adjusted for potential confounders if any significant differences were found between the study groups. To select significant variables, firstly under the GEE, the model-building strategy started by fitting a model containing all possible covariates in the data by considering exchangeable working correlation assumptions. To select the important factors related to the response variable, the backward selection procedure was used. This means that variables that did not contribute to the model based on the highest p-value were eliminated sequentially and each time a new model with the remaining covariates was refitted. The effect of the intervention on the outcome was measured using the odds ratio with a 95% confidence interval at a p-value less than 0.05 significant level.

### Ethical considerations

The study was carried out in line with the Helsinki Declaration. Ethical approval received from Jimma University, institute of Health Ethical Review Board (Reference Number JUIH/IRB 229/22). Written permission was taken from all relevant authorities in the Dessie and Kombolcha town zones. After ethical approval, the principal investigator communicated with Ethio Telecom to release three Subscriber Identity Module(SIM) cards that were used as behavioral intervention Sending Message Service (SMS). The participants were informed and written consent was obtained. Participants were offered a chance to withdraw from the study, and participation was entirely voluntary. If the woman is not educated and can not read the message, she is linked at recruitment with the nearest/trusted family member/husband who can read the message to her. If the woman has no mobile phone, but her husband/child/relative who lives in the house has a mobile phone she was linked with the mobile owner. Interviews were conducted in complete privacy and the confidentiality of study participants was kept.

## Results

### Socio-demographic and economic characteristics of participants

A total of 764 mothers participated in this study with a 97.4% response rate. Three hundred eighty-one (49.8%) and 383 (50.1%) of the respondents were controls and interventions respectively. The mean age of the respondents was 29.5 years (SD ± 4.4) with a minimum and maximum age of 19 and 42 years respectively. Three hundred fifty-three (92.6%) and 362(94.5%) of the respondents among controls and interventions respectively were married. One hundred sixty-seven (43.8%) and 185(48.3%) of the participants among controls and interventions were autonomous respectively. One hundred sixty-nine (44.3%) and 149(38.9%) of the participants among controls and interventions were secondary school education respectively. One hundred eighty-nine (49.6%) and 199(52%) participant controls and interventions were housewife occupations respectively. One hundred forty-six (38.3%) and 166(43.3%) of the participant controls and interventions were under poor wealth status respectively (Table 1).

**Table 1:** Socio-demographic and economic characteristics of participants in Dessie and Kombolcha cities, northeast Ethiopia, 2023 (N = 764).

Variables	Categories	Study group		Total(%)
		Control(%)	Intervention(%)	
Age	19–24	47(6.2)	49(6.4)	96(12.6)
	25–29	136(17.8)	150(19.6)	286(37.4)
	30–34	125(16.4)	135(17.7)	260(34.0)
	35+	73(9.6)	49(6.4)	122(16.0)
Women’s autonomy	Not autonomous	214(28.0)	198(25.9)	412(53.9)
	Autonomous	167(21.9)	185(24.2)	352(46.1)
Religion	Muslim	254(33.2)	229(30.0)	483(63.2)
	Christian	127(16.6)	154(20.2)	281(36.8)
Marital status	Married	353(46.2)	362(47.4)	715(93.6)
	Others	28(3.7)	21(2.7)	49(6.4)
Women’s education status	read and write	24(3.1)	17(2.2)	41(5.4)
	Primary	107(14.0)	164(21.5)	271(35.5)
	Secondary	169(22.1)	149(19.5)	318(41.6)
	College and above	81(10.6)	53(6.9)	134(17.5)
Husband’s educational status	Read and write	20(2.6)	9(1.2)	29(3.8)
	Primary	60(7.9)	95(12.4)	155(20.3)
	Secondary	175(22.9)	180(23.6)	355(46.5)
	College and above	126(16.5	99(13.0)	225(29.5)
Women occupation	Government employ	81(10.6)	67(8.8)	148(19.4)
	Private employ	35(4.6)	59(7.7)	94(12.3)
	House wife	189(24.7)	199(26.0)	388(50.8)
	Merchant	73(9.6)	54(7.1)	127(16.6)
	Unemployed	3(0.3)	4(0.5)	7(0.9)
Husband occupation	Government employ	142(18.6)	156(20.4)	298(39.0)
	Farmer	15(2.0)	2(0.3)	17(2.2)
	Private employ	131(17.1)	97(12.7)	228(29.8)
	Merchant	88(11.5)	124(16.2)	212(27.7)
	Unemployed	5(0.7)	4(0.5)	9(1.2)
Marital age	15–17	53(6.9)	55(7.2)	108(14.1)
	18–24	292(38.2)	265(34.7)	557(72.9)
	> = 25	36(4.7)	63(8.2)	99(13.0)
Family size	< = 4	278(36.4)	260(34.0)	538(70.4)
	>4	103(13.5)	123(16.1)	226(29.6)
Wealth Index	Poor	146(19.1)	166(21.7)	312(40.8)
	Middle	60(7.9)	50(6.5)	110(14.4)
	Rich	175(22.9)	167(21.9)	342(44.8)
Have health insurance	no	245(32.1)	259(33.9)	504(66.0)
	yes	136(17.8)	124(16.2)	260(34.0)
Sex of child	Female	188(24.6)	178(23.3)	366(47.9)
	Male	193(25.3)	205(26.8)	398(52.1)

### Obstetric characteristics of participants

Three hundred twenty-five (85.3%) and 339 (88.5%) of the participants among controls and interventions were multipara mothers respectively. One hundred eighty (47.2%) and 190(49.6%) of the participants among controls and interventions had less than 24 months birth to pregnancy interval respectively. Three hundred sixty-eight (96.5%) and 376(98.2%) of the participants among controls and interventions had live birth outcomes of pregnancy respectively. Three hundred thirty-five (87.9%) and 332 (86.6%) of the participants among controls and interventions had normal vaginal delivery respectively (Table 2).

**Table 2:** Obstetric characteristics of participants in Dessie and Kombolcha cities, northeast Ethiopia, 2023 (N = 764).

Variables	Categories	Study group		Total (%)
		Control (%)	Intervention (%)	
Number of pregnancies ever had	1	56(7.3)	44(5.8)	100(13.1)
	2–3	244(31.9)	267(34.9)	511(66.9
	> = 4	81(10.6)	72(9.4)	153(20.0)
Had abortion history	no	246(32.2)	295(38.6)	541(70.8)
	Yes	135(17.7)	88(11.5)	223(29.2)
Had stillbirth history	no	311(40.7)	325(42.5)	636(83.2)
	yes	70(9.2)	58(7.6)	128(16.8)
Birth to pregnancy interval	<12 months	93(12.2)	89(11.6)	182(23.8)
	12–23 month	87(11.4)	101(13.2)	188(24.6)
	24–36 month	122(16.0)	93(12.2)	215(28.1)
	>36 months	79(10.3)	100(13.1)	179(23.4)
Had ANC follow-up	no	19(2.5)	2(0.3)	21(2.7)
	yes	362(47.4)	381(49.9)	743(97.3)
ANC place	Health center	275(36.0)	205(26.8)	480(62.8)
	Hospital	16(2.1)	52(6.8)	68(8.9)
	Private health facility	34(4.5)	60(7.9)	94(12.3)
Health facility from home	Good near	93(12.2)	69(9.0)	162(21.2)
	Medium	241(31.5)	274(35.9)	515(67.4)
	Too far	47(6.2)	40(5.2)	87(11.4)
Place of delivery	Health center	207(27.1)	202(26.4)	409(53.5)
	Home	18(2.4)	2(0.3)	20(2.6)
	Hospital	62(8.1)	127(16.6)	189(24.7)
	Private health facility	94(12.3)	52(6.8)	146(19.1)
Outcome of pregnancy	IUFD	6(0.8)	3(0.4)	9(1.2)
	Live birth	368(48.2)	376(49.2)	744(97.4)
	Stillbirth	7(0.9)	4(0.5)	11(1.4)
Mode of delivery	Vacuum/forceps delivery	16(2.1)	15(2.0)	31(4.1)
	Normal vaginal delivery	335(43.8)	332(43.5)	667(87.3)
	C/S delivery	30(3.9)	36(4.7)	66(8.6)
Gestational age during delivery	<37 weeks	12(1.6)	42(5.5)	54(7.1)
	> = 37 weeks	369(48.3)	341(44.6)	710(92.9)

### Knowledge on early postpartum modern contraception

Concerning knowledge related to early postpartum contraception among participants 171(44.8%) control and 210(54.8%) intervention group had good knowledge on birth spacing and early postpartum contraception. Related to the timing of pregnancy can happen after the child’s 208(27.2%) control and 225(29.5%) interventions reported that starting from 45 days after childbirth pregnancy can happen if she is sexually active. One hundred fifty-four (20.2%) control and 228(29.8%) intervention group participants explained a woman can take the birth control method immediately after birth. One hundred forty (18.3%) controls and 243(31.8%) interventions reported a minimum birth interval to the health of the mother and the child was 2–3 years. One hundred fifty-four (20.2%) controls and 317(41.5%) interventions said that Mobile health messages help to improve knowledge of health (Table 3).

**Table 3:** Knowledge on early postpartum contraception of participants in Dessie and Kombolcha cities, northeast Ethiopia, 2023 (N = 764).

Variables	Categories	Study group		Total (%)
		Control (%)	Intervention (%)	
Time be pregnant after childbirth if she sexually practices	After 2 years	12(1.6)	23(3.0)	35(4.6)
	After 45 days	208(27.2)	225(29.5)	433(56.7)
	After 6 months	149(19.5)	94(12.3)	243(31.8)
	others	12(1.6)	41(5.4)	53(6.9)
Pregnancy can happen without seeing menstruation after childbirth	No	153(20.0)	179(23.4)	332(43.5)
	Yes	228(29.8)	204(26.7)	432(56.5)
A narrow birth interval has a negative health impact on the mother	No	39(5.1)	58(7.6)	97(12.7)
	Yes	342(44.8)	325(42.5)	667(87.3)
A woman can take birth control method immediately after birth	No	227(29.7)	155(20.3)	382(50.0)
	Yes	154(20.2)	228(29.8)	382(50.0)
Minimum birth interval to the health of the mother and the child	1 year	27(3.5)	25(3.3)	52(6.8)
	2–3 years	140(18.3)	243(31.8)	383(50.1)
	4–5 years	99(13.0)	186(24.3)	285(37.3)
	Do not know	32(4.2)	12(1.6)	44(5.8)
Mobile health messages help to improve knowledge of health	No	227(29.7)	66(8.6)	293(38.4)
	Yes	154(20.2)	317(41.5)	471(61.6)
A woman should take birth control method within 45 days after childbirth	No	102(13.4)	30(3.9)	132(17.3)
	Yes	279(36.5)	353(46.2)	632(82.7)
Time to take birth control method after childbirth to prevent early pregnancy	Within 48 hours of birth	108(14.1)	153(20.0)	261(34.2)
	Within 6 weeks of birth	151(19.8)	156(20.4)	307(40.2)
	After 6 months of birth	47(6.2)	47(6.2)	94(12.3)
	6 months to 1 year	22(2.9)	40(5.2)	62(8.1)
	Do not know	32(4.2)	8(1.0)	40(5.2)
Knowledge of EPP contraception	Poor knowledge	209(54.8)	173(45.2)	382(50.0)
	Good knowledge	171(44.8)	210(54.8)	382(50.0)

### Early postpartum contraceptive method adoption and fertility preference

**Fig 2:**
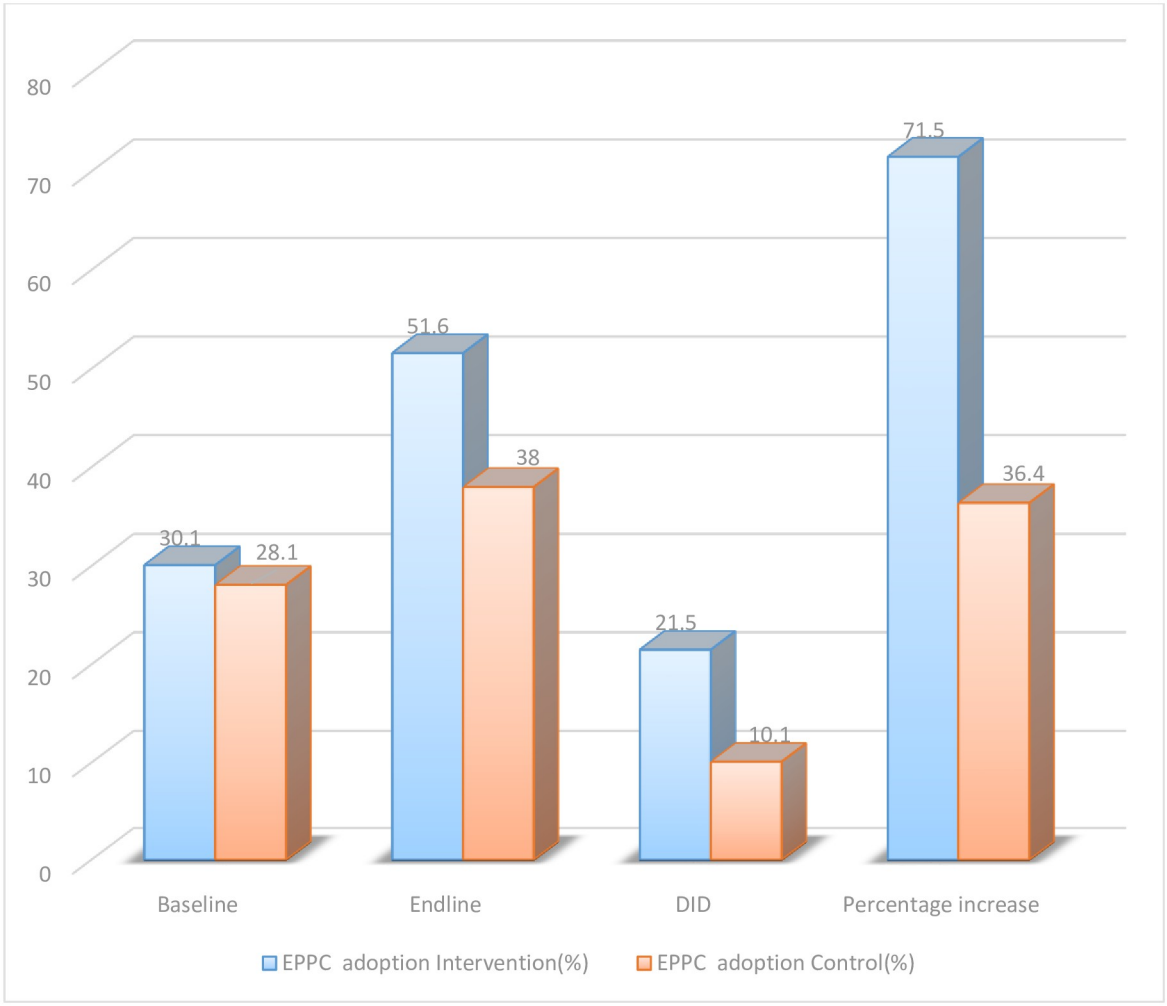
The difference in the difference of early postpartum contraception adoption among controls and intervention groups to the study effectiveness of mobile health intervention to improve early postpartum contraception adoption among mothers in Dessie and Kombolcha city zones, Northeast Ethiopia.

Two hundred fifteen (28.0%) and 217 (28.4%) of the participants among controls and interventions used contraceptive methods before this pregnancy respectively. Among these 107(28.1%) and 115(30.1%) of the participants were among controls and interventions taken during the early postpartum period respectively. Three hundred twenty-three (42.2%) and 315 (42.0%) of the participants among controls and interventions had family planning demand for spacing. Three hundred (78.7%) and 296 (77.3%) of the participants among controls and interventions had sexual practice after childbirth respectively (Table 4). One hundred forty-six (38.0%) and 198(51.6%) of the participants among controls and interventions took and used modern contraceptive methods after childbirth respectively (Fig 2).

**Table 4:** Early postpartum contraceptive method adoption and fertility preference of participants in Dessie and Kombolcha city zones, northeast Ethiopia, 2023 (N = 764).

Variables	Categories	Study group		
		Control (%)	Intervention (%)	Total (%)
Used contraceptive method before this pregnancy	No	167(21.9)	166(21.7)	333(43.6)
	Yes	214(28.0)	217(28.4)	431(56.4)
Time is taken contraceptive method before this pregnancy.	Within 6 weeks after childbirth	107(14.0)	115(15.1)	222(29.1)
	6 weeks to 6 months childbirth	66(8.6)	55(7.2)	121(15.8)
	After 6 months of childbirth	35(4.6)	40(5.2)	75(9.8)
	After 9months	4(0.5)	2(0.3)	6(0.8)
	Others	8(1.0)	1(0.1)	9(1.2)
Future fertility preference	Want no more	37(4.8)	46(6.0)	83(10.9)
	Undecided	44(5.8)	83(10.9)	127(16.6)
	Want but time not decide	118(15.4)	110(14.4)	228(29.8)
	Want later	161(21.1)	122(16.0)	283(37.0)
	Want more soon	21(2.7)	22(2.9)	43(5.6)
A woman should take birth control method within 45 days after childbirth	No	102(13.4)	30(3.9)	132(27.3)
	Yes	279(36.5)	353(46.2)	632(82.7)
Take and use any modern contraceptive after childbirth	No	235(30.8)	185(24.2)	420(55.0)
	Yes	146(19.1)	198(25.9)	344(45.0)
Have sexual practice after birth	No	81(10.6)	87(11.4)	168(22.0)
	Yes	300(39.3)	296(38.7)	596(78.0)

### Intervention effectiveness to enhance adoption of early postpartum modern contraceptive method: Generalized Estimating Equation(GEE) model

The GEE, model building strategy was started by fitting a model containing all possible covariates in the data by considering exchangeable working correlation assumptions. To select the important factors related to the effectiveness of mHealth intervention, the backward selection procedure was used. After fitting the model, covariates with the largest p-value of the Wald test were removed and refitted the model with the rest of the covariates sequentially. Then, women’s occupation, mode of delivery, women educational status, husband’s educational status, religion, complications during delivery, knowledge about early postpartum contraception, wealth status, women’s autonomy, intention to adopt during pregnancy, place of delivery and future fertility preference were the covariates excluded from the model with Wald test p-value for the given covariates were large (P-value > 0.05).

The QIC values of the full model and reduced models were 4100.0609 and 4098.4137 respectively. Then it turned out that the model with mHealth intervention, parity, sexual practice after childbirth, previous experience of early postpartum contraception, and status of child as covariates was the most parsimonious model (Table 5). Finally, as customary, a comparison of empirical and model-based standard errors for the parameter estimates obtained based on the given working correlation assumptions was performed using selected covariates. The correlation structure with the model-based and empirical standard errors are closest to each other and is referred to be the best assumption correlation structure.

**Table 5:** Parameter estimates for the GEE model to the study on the effectiveness of mHealth intervention to enhance uptake of early postpartum contraceptive method among women in Dessie and Kombolcha Zones, North-East Ethiopia.

Effects	Category	*Adjusted Odds ratio*	Estimates (S.e)	*P-value*	95% conf.int
Parity	Primipara	1			
	multipara	1.7	0.4	0.02	1.069–2.695*
Had abortion history	no	1			
	Yes	0.7	0.1	0.06	0.525–1.02
Had sexual practice after childbirth	no	1			
	yes	1.9	0.3	0.001	1.378–2.887**
Had ANC follow-up	no	1			
	yes	1.7	0.9	0.282	0.623–5.052
Headofhousehodrecod	Female-headed	1			
	Male headed	0.5	0.1	0.057	0.300–1.017
Status of child	died	1			
	alive	3.7	2.4	0.04	1.034–13.353*
Women occupation	Housewife	1			
	Other paid work	0.7	0.1	0.16	0.437–1.153
Previous experience of EPP Contraception	Birth to 6 weeks	1			
	6weeks to 6month	0.5	0.1	0.01	0.358–0.912**
	After 6months	0.6	0.1	0.182	0.402–1.188
	after_9months	0.4	0.3	0.364	0.075–2.589
Knowledge_EPPC	Poor knowledge	1			
	Good knowledge	0.7	0.1	0.097	0.570–1.047
Had Intention during pregnancy to uptake	no	1			
	yes	0.8	0.1	0.206	0.612 1.111
mHealth intervention	no	1			
	Yes	1.6	0.2	0.0001	1.249–2.123***

*** p<0.01,

** p<0.05,

* p<0.1,

1 reference category

After controlling all other variables in the model, the odds of uptake of the postpartum modern contraceptive method during the early postpartum period among mothers who were in the mHealth intervention was 1.6 times (AOR: 1.6 (95% CI: 1.249–2.123) higher than compared to those mothers in the control group. This means that the probability of adoption of early postpartum modern contraception of mothers with mHealth intervention was 60% higher with mothers in intervention group as compared with the control group.

There is also a strong association between previous experience of EPP Contraception and the adoption of modern contraceptive methods during the early postpartum period among mothers. This implies that, after adjusting all other predictor variables in the model, the estimated odds ratio of using the early postpartum model contraceptive method was 50% (AOR: 0.5(95% CI: 0.358–0.912) less likely among mothers who took EPP contraception after 6 weeks postpartum than mothers who took before 6 weeks of postpartum previously.

Mothers who had alive newborns were more likely (AOR: 3.7, 95%CI: 1.034–13.353) to receive an early postpartum contraceptive method than those mothers whose newborn status died. This means that the probability of early adoption of the postpartum modern contraceptive method was 3.7 times more likely than the mothers who faced the death of their newborn. Parity status of the mother was also another influential predictor variable, for the early adoption of postpartum modern contraceptive methods to mothers. The odds of early uptake of modern contraceptive methods during the postpartum period were 1.7. Times higher among multipara mothers (AOR: 1.7, 95% CI: 1.069–2.695) than primigravida mothers.

## Discussion

The presented study provides valuable insights into the demographic and obstetric characteristics of postpartum women, shedding light on factors that may influence their reproductive health choices. A comparative discussion with other relevant studies allows for a broader understanding of these findings.

In this study, 47.2% of control group participants and 49.6% of intervention group participants had a birth-to-pregnancy interval of less than 24 months. This aligns with results from previous literature [38, 39], highlighting the challenge of short birth intervals in various populations. Short intervals are associated with increased health risks for both mothers and infants, emphasizing the importance of addressing this issue in family planning programs.

The discrepancy in the incidence of delivery complications is noteworthy, with 18.8% of control group participants and 28.3% of intervention group participants facing complications. This variation may be attributed to differences in healthcare settings, socio-economic factors, or access to quality maternal care. A comparison underscores the importance of addressing delivery complications as a potential barrier to optimal postpartum family planning outcomes [40].

The prevalence of contraceptive use before the current pregnancy was notable, with 28.0% and 28.4% among controls and interventions, respectively. This aligns with findings from [41, 42], where a similar percentage of participants reported utilizing contraceptives before their latest pregnancies. This consistency suggests a common trend in pre-pregnancy contraceptive practices across diverse populations.

Furthermore, the study indicates that a significant proportion of participants in both groups initiated contraceptive use during the early postpartum period before the intervention, with 28.1% and 30.1% among controls and interventions, respectively. This closely resembles the findings in [43, 44], emphasizing the importance of addressing family planning needs promptly after childbirth. These results collectively underscore the need for interventions that target the early postpartum period to maximize the impact on contraceptive uptake.

While 42.2% and 42.2% of participants in controls and interventions expressed a demand for spacing, 4.8% and 6.0% had a demand for limiting among control and intervention groups, respectively. A comparative analysis with [45, 46] reveals similar trends, suggesting that the preference for spacing over limiting is a common pattern across different populations. The study shows that a significant proportion of participants resumed sexual practices after childbirth (78.7% in controls and 77.3% in interventions) is consistent with findings from [47–49]. This consistency in postpartum sexual practices indicates the relevance of integrating family planning services into postpartum care to address the contraceptive needs of sexually active individuals.

The uptake of modern contraceptive methods post-childbirth is a crucial aspect of family planning. The study reports that 38% and 51.6% of participants in controls and interventions, respectively, used modern contraceptive methods after childbirth during the early postpartum period. This finding is consistent with other interventional studies [14, 50]. This suggests that the higher percentage in the intervention group might be attributed to specific interventions or programs implemented, emphasizing the need for targeted strategies to enhance modern contraceptive adoption.

The odds of adopting postpartum modern contraceptive methods during the early postpartum period were 1.6 times higher among mothers who received the mHealth intervention compared to those in the control group (AOR: 1.6, 95% CI: 1.249–2.123). This finding is consistent with other literature [51, 52]. This implies a 60% increased probability of early adoption of contraception among mothers exposed to the mHealth intervention.

There was a significant association between previous experience of early postpartum contraception and the likelihood of adopting modern contraceptive methods. Mothers who initiated contraception after 6 weeks postpartum were 50% less likely to adopt modern methods compared to those who started before 6 weeks (AOR: 0.5, 95% CI: 0.358–0.912), after adjusting for other predictor variables. The findings are consistent with previous literature [53, 54].

The status of the newborn emerged as a crucial factor, with mothers having a live newborn being 3.7 times more likely to adopt early postpartum modern contraceptive methods compared to those whose newborns died (AOR: 3.7, 95% CI: 1.034–13.353). This finding is with other studies [55]. Additionally, multipara mothers exhibited 1.7 times higher odds of early contraceptive uptake compared to primigravida mothers (AOR: 1.7, 95% CI: 1.069–2.695). This finding is consistent with other studies [56, 57].

### Limitations of the study

Cultural, regional, access to telecom infrastructure, or healthcare system differences could limit the applicability of the results to other contexts. Participants may provide responses that they perceive as socially desirable, potentially leading to an overestimation of positive behaviors.

## Conclusion

This study findings demonstrated that the potential effectiveness of mobile health interventions in promoting timely contraceptive uptake during early postpartum period. The mobile health intervention, combined with factors such as timing of previous contraceptive initiation, newborn status, and maternal parity, significantly enhances the likelihood of early contraceptive adoption. These nuanced insights provide a strong foundation for developing targeted health interventions and policies aimed at improving early postpartum contraception. Policymakers and programmers should expand and enhance mobile health interventions, integrating technology to disseminate information, provide support, and facilitate access to family planning services during early postpartum period. Minster of health with regional health bureaus should establish monitoring and evaluation mechanisms to assess the ongoing effectiveness and long-term impact of interventions. Health care providers should targeted support and counseling should be provided to mothers who have experienced neonatal loss, given the significant influence of the newborn’s status on contraceptive adoption. Sexual health education and services should be integrated into early postpartum care to address both reproductive and sexual health needs. Additionally, researchers should evaluate the cost-effectiveness of mobile health interventions and explore the potential of mobile call interventions in other health programs for future digital generations.

## Supporting information

S1 Checklist Human participants research checklist. (DOCX)

S1 File Research protocol to the study. (DOCX)

S2 File Data set of the study. (SAV)

S3 File Intervention packeges and schedule to the study. (DOCX)

## Abbreviations

ANC: Antenatal care
EDHS: Ethiopia Demographic, and Health Survey
EPPMFP: Early Post-Partum Modern Family Planning
FP: Family Planning
IMHI: Interactive Mobile Health Intervention
mHealth: mobile Health
ODK: Open Data Kit
SDG: Sustainable Development Goals
SMS: Sending Message Service
WHO: World Health Organization
