# Supplementary material for: Effectiveness of an Interactive Mobile Health Intervention (IMHI) to enhance the adoption of modern contraceptive methods during the early postpartum period among women in Northeast Ethiopia: A cluster Randomized Controlled Trial (RCT)
Source: PLoS One. 2024 Nov 14;19(11):e0310124. doi: 10.1371/journal.pone.0310124 (PMC11563424; doi:10.1371/journal.pone.0310124)
Supplement: S1 File — (DOCX) [file pone.0310124.s002.docx]

**The Effectiveness of an Interactive Mobile Health Intervention (IMHI) to enhance the Adoption of Modern Contraceptive Methods during the Early Postpartum Period among Women in Dessie and Kombolcha, Northeast Ethiopia: A Cluster Randomized Controlled Trial (RCT): Research protocol**

Niguss Cherie^1 2*^, Muluemebet Abera Wordofa^1^, Gurmesa Tura Debelew^1^

**^1^** Population and Family Health Department**,** Faculty of Public Health**,** Institute of Health, Jimma University, Jimma, Ethiopia, ^2^Reproductive and Family Health Department, School of Public Health, College of Medicine and Health Sciences, Wollo University, Dessie, Ethiopia

**Email Address**:

Niguss [Cherie (MPH/RH, Assistant Professor): nigucheru@gmail.com](mailto:Cherie%20(MPH/RH,%20Assistant%20Professor):%20nigucheru@gmail.com)

Muluemebet Abera Wordofa (Associate professor, PhD): [mulu_abera.ts2009@yahoo.co](mailto:mulu_abera.ts2009@yahoo.co)m

Gurmesa Tura Debelew (Professor, PhD): gurmesatura@gmail.com

***Corresponding author:** Niguss Cherie

**^1^** Population and Family Health Department**,** Faculty of Public Health**,** Institute of Health, Jimma University, Jimma, Ethiopia, ^2^Reproductive and Family Health Department, School of Public Health, College of Medicine and Health Sciences, Wollo University, Dessie, Ethiopia

**Email- [nigucheru@gmail.com](mailto:nigucheru@gmail.com)**

**Mobile: +251910749743**

# Summary

**Background**: Women in the early postpartum period face substantial unmet needs in contraception to encourage birth intervals and reduce unintended pregnancies. The widespread ownership of mobile devices offers an opportunity to employ mobile health strategies for enhancing communication between healthcare providers and mothers across various levels. But, little is nown about effectiveness of mHealth intervention to enhance early adoption of contraceptives methods after childbirth in developing countries.

**Objectives:** This study will be aimed to determine the effectiveness of mobile health intervention to enhance uptake of modern contraceptive method in early postpartum period in Dessie and Kombolcha town zones, Northeast Ethiopia.

**Methods**: The research will be carried out in Dessie and Kombolcha city zones located in the Amhara region of Northeast Ethiopia during January 15 to Jun 15, 2023. Pregnant women with a confirmed gestation of 24-28 weeks will be enrolled to baseline assessment , then the intervention will be started at 30 weeks and followed upto 45 days postpartum period. The study will be employed a cluster randomized control trial involving 784 participants (392 controls and 392 intervention). The intervention group received a new mobile health intervention in addition to the existing healthcare practices, while the control group will be solely adhered to the current healthcare practices. Data will be collected by Open Data Kit (ODK) and exported to STATA.17 for analysis. Marginal model Generalized Estimating Equations (GEE) through the application of an exchangeable working correlation will be applied. Despite the random allocation of participants to study groups, estimates will be adjusted for potential confounders. The effect of the intervention on the outcome will be measured using the odss ratio with a 95% confidence interval at p-value less than 0.05 significant level.

**Trial registration**: Protocol Registration and Results System (PRS) Clinical Trial Registry, www. ClinicalTrials.gov, ID: ClinicalTrials.gov ID: NCT05666037. Registered on December 23, 2022.

**Keywords**: - Mobile health, early postpartum contraception, randomized control trial, northeast Ethiopia.

#

## BACKGROUND

Early postpartum contraceptive adoption is defined as women who have ever used any kind of modern birth control method within the first six weeks after they gave birth(1,2). Early Postpartum family planning contributes to the reduction of narrow birth intervals, and unplanned and unintended pregnancy further contributes to the reduction of maternal and newborn death. Postpartum women have among the highest unmet needs for family planning to promote longer birth intervals (3).

Evidence shows that short birth intervals increase the danger of maternal, newborn, neonatal, and associated under-5 mortality(4); and is related to magnified risk of preterm birth, low birth weight, stunting, and skinny youngsters (5–7).To reduce the danger of adverse maternal, perinatal, and neonatal outcomes, the World Health Organization (WHO) recommends a minimum of 24-36 months interval between delivery and the later gestation(8,9). Early postpartum contraception is a well-tried and cost-efficient intervention to stop each maternal and newborn death by reducing the short birth intervals, the number of abortions, and the proportion of births at high risk(10).

The early post-partum period provides a unique opportunity to meet the reproductive health needs of women particularly the need for contraception after childbirth. The timing of the return of fertility after childbirth is variable and unpredictable. Some women resume ovulation and menstruation as early as 28 days post-delivery(11). Consequently, gaps exist in meeting the demand for contraception among women of reproductive age, particularly in the early post-partum period(12).

The promotion of early postpartum contraception in countries with high birth rates has the potential to avert 32% of all maternal deaths and nearly 10% of childhood deaths(13). Early postpartum Contraceptive prevalence is still relatively low (38.5%) %) in Ethiopia, and the unmet need (25%)(14). Nearly half (47%) of postpartum women have short (<23 months) birth-to-pregnancy intervals in Ethiopia(15).

The Ethiopian government is putting great efforts into programmatic and policy initiatives in place to increase access to and utilization of early postpartum contraceptive methods through the rapid expansion of primary health care facilities, massive training of midwives, and free provision of family planning services(16). However, the level of early postpartum contraceptive method uptake is still unacceptably low. Despite the national efforts Ethiopia continues to have an unmet contraceptive need and a high rate of maternal morbidity and mortality associated with pregnancy, childbirth, and postpartum(17).

Women received guidance and counseling concerning narrow birth prevention throughout the antepartum and immediate postnatal period(18). However, once discharged, most women don't come to health facilities for follow-up visits for birth prevention service providers(19). Because of the high proportion of postpartum women lost to follow-up at health facilities the potential for postnatal narrow birth prevention has not been realized(20). The growth and access for mobile phones and mobile services and unexampled increase in mobile penetration are anticipated to facilitate the use of mHealth initiatives in resource-restricted settings (21,22). Extending the reach of the healthcare system, mHealth is intended to function as a cue to action and boost communications to support healthcare behavior amendment(23). High mobile phone ownership presents a chance to utilize mHealth approaches to push behavior amendment and reminder intervention on maternal and child health care in the community(24,25).

There is growing evidence showing that ordinarily utilized mobile health solutions (mHealth) like Sending Message Service(SMS) used to improve health service delivery processes and health outcomes within the developed world(26). However, no evidence demonstrates the effectiveness of mHealth interventions on key maternal and child health service outcomes in Ethiopia.

This study will be aimed to incorporate the family planning counseling guideline with the mobile short message with the hypothesis that such intervention would be effective in enhancing uptake of contraceptive methods during early postpartum period. Thus, the target of the planned study will be to intervene the effectiveness of mobile health intervention to enhance early postpartum modern contraceptive method adoption among mothers in Dessie and Kombolcha city zones, Northeast Ethiopia. The findings of this study will be expected to contribute the existing knowledge gap, to understand the possible technology-based interventions for behavior change in the community, and act accordingly. Additionally, the findings will be used as baseline information for improving healthcare services to policymakers, reproductive health programmers, program implementers, NGOs, local health planners, and healthcare providers.

# METHODS AND MATERIALS

## Study area, design, and period

The study will be conducted in the Dessie and Kombolcha city zones in Amhara regional state, Northeast Ethiopia. Dessie is the administrative town of the south Wollo zone, which is situated 401 KM from Addis Ababa to the north. Dessie city is split into 5 sub cities with 22 kebeles and has 2 governmental hospitals and 8 health centers. Based on population projection for 2023 more than 470,000 residents population with an estimated 21, 620 pregnant women in Dessie town. Kombolcha town is 30 km from Dessie city and 375 km from Addis Ababa is an industrial zone and dry port in northeast Ethiopia. There are more than 350,000 resident populations with 16,100 estimated pregnant women in Kombolcha town. It is divided into 5 sub cities with 19 kebeles and has one governmental hospital and five health centers (27). Cluster randomized control trial study will be conducted from January 15 to Jun 15, 2023.

## Population and eligibility

All post-partum women in the study area will be taken as the source population. First census will be conducted to identify eligible pregnant women and a baseline study was done. All eligible pregnant women based on World Health Organization pregnancy screening eligibility criteria at 30 weeks gestational age will be included in the intervention and control group then followed up to 6 weeks postpartum.

## Sample size determination and sampling procedures

The sample for the study will be determined using the assumption of superiority trial design using STATA 17 to demonstrate the superiority of a new intervention compared to the existing early postpartum contraceptive adoption increased from 38.5% to 48.5% (14) . The following assumptions will be used to calculate the sample size. Error probabilities (0.05), Power (80%), the ratio of several clusters (1), Effect size proportion to control (38.5%), Effect size proportion to experiment (48.5%), and intra-class correlation (10%). The total sample size with a 10% non-response rate of study subjects will be 784(392 interventions and 392 controls). A cluster sampling technique will be applied. First, clusters (Kebeles) will be randomly selected and census will be conducted to identify and register pregnant women based on eligibility criteria. All registered eligible pregnant women in the selected clusters (Kebeles) will be included in the baseline study. After baseline data collection clusters will be assigned randomly as intervention or control groups (Fig 1).

Dessie and Kombolcha City zones

Randomization

Kombolcha city zone

19 clusters (kebeles)

Dessie city zone

22 clusters (Kebeles)

Simple Random

Simple Random

10 clusters (Kebeles)

Number of estimated pregnant women (10,810)

10 clusters (Kebeles)

Number of estimated pregnant women (8,050)

Assessment for Eligibility

- Gestational age>28 weeks
- Having mobile phone
- Willing to participate

Target Enrolment n_2_ = 392 pregnant mothers were registered with phone number and ID number

Census

Census

Target Enrolment n_1_ = 392 pregnant mothers were registered with phone number and ID number

Enrolment

Baseline Data collection (392)

- House to House Survey

Baseline Data collection (392)

- House to House Survey

Baseline data collection

Fig 1: Consolidated Standards of Reporting Trials (CONSORT) Diagram depicts the sampling method and allocation of study units, assessment of eligibility criteria to include study participants to the effectiveness of mHealth intervention to improve adoption of contraceptive method during early postpartum period in Dessie and Kombolcha city zones, Northeast Ethiopia, 2023.

# Description of the intervention

# The mobile health intervention for enhancing the early adoption of modern contraceptive methods among postpartum mothers will bea behavior change and reminder initiative designed to enhance maternal and child health outcomes. The study initially will divided participants into two groups through random cluster assignment. The intervention group will be received a series of text messages (SMS) promoting behavioral change and encouraging the uptake of modern contraceptive method during the early postpartum period. This communication will be spanned four months, covering 90 days prenatally and 42 days postpartum. In contrast, the control group will be relied solely on routine healthcare providers at health facilities without any mobile-based interventions.

# During the study, participants in the intervention group will be received one text message every two weeks, with the frequency increasing to daily messages for the 42 days following delivery. Trained female health workers, proficient in the local language will be delivered the intervention. Each participant will be received a total of eight SMS over the four-month period. In cases where women will be possessed mobile phones but lacked formal education or the ability to read the messages, they will be connected with their husbands or nearest family members, who would read the messages to them.

# Intervention module development

Researchers developed early postpartum family planning intervention mobile health messages from culturally congruent family planning behavior change framework, and national and WHO family planning guidelines((27,28). The messages consist of a congratulatory message, counseling on maternal and neonatal health needs, the dangers of narrow birth intervals, time of fertility return after childbirth, and planning future pregnancies. All messages will be developed in English and later translated into the local language Amharic. Additionally, researchers will be gathered feedback from experts and used findings to further refine the behavior change intervention messages. The intervention schedule and mobile health package messages are annexed in Table 2

# Recruitment and participant timeline

Pregnant women with 26-28 weeks of gestation(based on WHO eligibility criteria) will be recruited and baseline data will be collected at selected clusters. Women who will be provided informed consent will be asked to complete a post-consent eligibility assessment including access to a mobile phone, willingness to participate in the follow-up study and to receive health messages on their mobile phone. Women who meet these eligibility criteria were enrolled in the study and will be administered the baseline interview. Baseline assesment and recruitment will be done among pregnant women with gestational age of 24-28 weeks of gestation and the intervention will be started at 30 weeks of gestation and continued up to 6 weeks of the postpartum period for 4 months. Four intervention worker female nurses will be recruited and trained to do the intervention based on the protocol. After this end, data will be collected from both the intervention and control groups.

**Strategies to mantain intervention fidelity**

The strategies to use to maintaine intervention fidelity will be comprehensive training sessions for individuals will be delivered the intervention, use training manuals, protocols, develope scripts, timing guidelines, and instructions for handling common issues that may arise during delivery. regular supervision and monitoring visits will be conducted to observe the intervention delivery and provide feedback.

## Assessing whether the intended content reached the target audience

## The investigators will be employed various assessment techniques to determine whether the intended content reached the audience. They investigators will use checklists and survey to gather information on participants' reception and understanding of the content. Self-report measures from implementers were used to capture their adherence to the intervention procedures and any challenges faced. Additionally, results from different groups were compared to assess if variations in fidelity impacted the intervention's effectiveness.

## Randomization to intervention or control group assignment

There is a 30 km buffer zone between Dessie and Kombolcha towns to prevent information contamination of the intervention. After baseline data collection intervention and control groups were allocated through stratified randomization of clusters. The kebeles in Dessie and Kombolcha cities were identified as clusters. Clusters were stratified based on the average number of pregnant women served per month and geographic location. Within each stratum, randomly assign clusters to either the intervention group or the control group using a computer-generated randomization sequence to ensure the randomization process was unbiased to minimize confounding factors and ensure a balanced distribution of health facilities across intervention and control groups, enhancing the reliability and validity of the study results. Outcome assessors were blinded to the allocation to prevent assessment bias.

## **Data collection tools and procedures**

Data will be collected by using pre-tested interviewer-administered structured questionnaires adapted from different literatures(2,14,29–31). All the questionnaires will be prepared in English, then translated into the local language Amharic, and translated to English to check their consistency. A form on the Open Data Kit (ODK) will be created, and data will be collected by the ODK Collect tool, with the aggregation data subsequently compiled on the KOBO Toolbox. Participant recruitment and baseline data collection will be done by eight trained college-completed nurses. End end-line survey will be conducted by eight female nurses well familiar with local geography and who were not involved in recruitment, baseline data collection, and intervention process. Before actual data collection, census will be conducted and a list of eligible pregnant women with important contact and follow-up addresses was obtained from selected clusters. Then, a specific identification number (code) will be given to all the registered pregnant women to avoid identifiers and to link the data during the intervention and follow-up. Following this actual baseline data will be collected from home to home in the community and endline data will be collected after 45 days of childbirth.

## Data quality assurance

Training for data collectors and pretests will be conducted. Intra-variability of interviewers will be tested by comparison information collected by the supervisors and also the data collectors. Four Master of Public Health (MPH) holders will be recruited and supervised the overall data collection process together with the principal investigator. When inconsistencies appear measure will be taken. The principal investigator and supervisors will be closely supervised activities throughout the intervention and study period. In addition, the PI and study supervisors will monitor adherence to check intervention protocol and data collection processes. The research team will be closely monitored for messages will be delivered with success. The team conjointly will be followed the successful completion of the intervention and dropouts among the study population.

## Data processing and analysis

Data from ODK will be exported to STATA 17. Descriptive and summary statistics will be done. In clustered data, observations are usually taken from the same unit, and thus this information forms a cluster of correlated observations. The marginal model Generalized Estimating Equations (GEE) will be done by using STATA 17. This model is preferred to avoid the clustering effects as the factors exist at different levels and violate the assumption of independence for the ordinary logistic regression. Although the participants will be randomly allocated to study groups, estimates will be adjusted for potential confounders if any significant differences were found between the study groups. To select significant variables, firstly under the GEE, model building strategy will be started by fitting a model containing all possible covariates in the data by considering exchangeable working correlation assumptions. In order to select the important factors related to the response variable, the backward selection procedure will be used.This means that variables that will not contribute to the model based on the highest p-value will be eliminated sequentially and each time a new model with the remaining covariates will be refitted. The effect of the intervention on the outcome will be measured using the odss ratio with a 95% confidence interval at p-value less than 0.05 significant level.

## Operational definitions

**Early postpartum contraceptive method use**: Early postpartum contraception is outlined as women who have ever used any kind of modern birth control technique at intervals the first six weeks when she gave birth(32). If the respondent answers yes it was coded as "1" and if not coded as "0"

**Intention to uptake early postpartum modern contraceptive method use**: Ten questions with a 5 Likert scale will be used to measure the intention of pregnant women to uptake early postpartum modern family planning. Respondents who scored above or equal to the mean/median value were considered as having intention to uptake, while those who scored below the mean/median value were considered as having no intention to uptake early post-partum modern family planning methods(33).

**Mobile health (mHealth):** Mobile health (mHealth) refers to the employment of wireless, moveable data and Communication Technologies (ICT) to support health and health care. For this study, mobile health includes sending message service (SMS) on early post-partum modern contraceptive method adaoption for behavior change intervention and reminde(31,34,35).

**Women autonomy**: We use 23 items applied considering the three categories namely decision-making autonomy, movement autonomy, and financial autonomy. Principal component analysis method with a fixed number of factors for measuring women's autonomy in the context of developing countries. Those will have mean/median and above values will be taken as autonomous (36).

**Wealth index:** We use 19 items applied considering the urban wealth assessment tool. Principal component analysis method with a fixed number of factors for measuring wealth index in the context of developing countries. Then categorized as rich, middle and poor based on percentile value of the score(37,38).

**Ethics approval and consent to participate**

**This study will be carried out in line with the Helsinki Declaration. The actual data collection will be carried out after getting ethical approval from the Ethical Review Committee of Jimma University, institute of Health Ethical Review Board.** Written permission will be given to all relevant authorities in the Dessie and Kombolcha town zones. After ethical approval, the principal investigator communicated with Ethio Telecom to release three sim cards that will be used as behavioral intervention Sending Message Service (SMS). The participants will be informed about the aim and purpose of the study, the importance of their participation, and their rights, and informed consent was obtained. Participants will be offered a chance to withdraw from the study, and participation was entirely voluntary. If the woman is not educated and can not read the message, she will be linked at recruitment with the nearest/trusted family member/husband who can read the message to her. If the woman has no mobile phone, but her husband/child/relative who lives in the house has a mobile phone she will be linked with the mobile owner. Interviews will be conducted in complete privacy and confidentiality and anonymity of study participants will be kept.

**References**

1. Cleland J, Shah IH, Daniele M. Countries : Program Implications and Research Priorities Interventions to Improve Postpartum Family Planning in Low- and Middle-Income Countries : Program Implications and Research Priorities In the early international family hospital-based postpartum con. 2015;46(4).

2. Tool D. A guide to family planning.

3. Approaches P. Increasing Family Planning Uptake Among Postpartum Women in Nigeria. 2020;(September 2018):1–5.

4. Collins F, Pardee FS, Fink G, Kuhn R, Studies D. Birth Spacing and Child. 2020;6:347–71.

5. Darmstadt GL, Pepper KT, Ward C, Mehta KM, Bentley J, Rangarajan A, et al. Impact of the Ananya program on reproductive , maternal , newborn and child health and nutrition in Bihar , India : early results from a quasi- experimental study. 2020;10(2):1–18.

6. Hackett K, Lafleur C, Nyella P, Ginsburg O, Lou W, Sellen D. Impact of smartphone-assisted prenatal home visits on women ’ s use of facility delivery : Results from a cluster-randomized trial in rural Tanzania. 2018;1–20.

7. Srikantiah S, Mahapatra T. workers to promote reproductive , maternal , randomized controlled Trial in Bihar , India. 2019;9(2).

8. Report M. Postpartum Family Planning Technical Consultation Meeting Report. 2006;(November).

9. Starbird E. Healthy Timing and Spacing of Pregnancy : Reducing Mortality Among Women and Their Children. 2019;7:211–4.

10. INTEGRATED FAMILY PLANNING PROGRAM ( IFPP ). 2021;(Dhs 2011):2011.

11. Kanakuze CA, Dan KK, Musabirema P, Pascal N. Factors associated with the Uptake of Immediate Postpartum Intrauterine Contraceptive Devices ( PPIUCD ) in Rwanda : A Mixed Methods Study. :1–15.

12. Pfitzer A, Lathrop E, Bodenheimer A, RamaRao S, Christofield M, MacDonald P, et al. Opportunities and challenges of delivering postabortion care and postpartum family planning during the covid-19 pandemic. Glob Heal Sci Pract. 2020;8(3):335–43.

13. Atnafu A, Bisrat A, Kifle M, Taye B, Debebe T. Original article Mobile health ( mHealth ) intervention in maternal and child health care : Evidence from resource-constrained settings : A review.

14. Tafere TE, Afework MF, Yalew AW. Counseling on family planning during ANC service increases the likelihood of postpartum family planning use in Bahir Dar City Administration , Northwest Ethiopia : a prospective follow up study. 2018;1–9.

15. Ali M, Farron M, Dilip TR, Folz R. Assessment of family planning service availability and readiness in 10 African countries. Glob Heal Sci Pract. 2018;6(3):473–83.

16. MOH, Sector H, Plan T. Health Sector Transformation Plan, 2020.

17. CSA, Demographic M, Survey H, Indicators K. No Title. 2019.

18. Zimmerman LA, Yi Y, Yihdego M, Abrha S, Shiferaw S, Seme A, et al. Effect of integrating maternal health services and family planning services on postpartum family planning behavior in Ethiopia : results from a longitudinal survey. 2019;1–9.

19. Tafere TE, Afework MF, Yalew AW. Does antenatal care service quality influence essential newborn care ( ENC ) practices ? In Bahir Dar City Administration , North West Ethiopia : a prospective follow up study. 2018;1–8.

20. Id BW, Mosisa G, Etafa W, Mulisa D, Tolossa T, Fetensa G, et al. Postpartum modern contraception utilization and its determinants in Ethiopia : A systematic review and meta-analysis. 2020;1–21.

21. Abejirinde IO, Ilozumba O, Marchal B, Zweekhorst M, Dieleman M. Mobile health and the performance of maternal health care workers in low- and middle-income countries : A realist review. 2018;

22. Balakrishnan R, Gopichandran V, Chaturvedi S, Chatterjee R. Continuum of Care Services for Maternal and Child Health using mobile technology – a health system strengthening strategy in low and middle income countries. BMC Med Inform Decis Mak [Internet]. 2016;1–8.

23. Nasution LA, Tutik R, Hariyati S. Mobile Health Application in Implementation of Maternity Nursing Care : Literature Review Studi Literatur : tentang Implementasi Aplikasi “ Mobile Health ” di Pelayanan Keperawatan Maternitas. 1(February 2018).

24. Atnafu A, Otto K, Herbst CH. The role of mHealth intervention on maternal and child health service delivery : findings from a randomized controlled field trial in rural Ethiopia. 2017;

25. Modi D, Dholakia N, Id RG, Id SV, Id KD, Id SS, et al. mHealth intervention “ ImTeCHO ” to improve delivery of maternal , neonatal , and child care services — A cluster-randomized trial in tribal areas of Gujarat , India. 2019;1–24.

26. Shiferaw S, Workneh A, Yirgu R, Dinant G, Spigt M. Designing mHealth for maternity services in primary health facilities in a low-income setting – lessons from a partially successful implementation. 2018;9:1–15.

27. Huff MB. Family Planning: A Global Handbook for Providers. J Pediatr Adolesc Gynecol. 2009;22(2):135.

28. Ministry of Health. Ethiopia. National Guideline for Family Planning Services in Ethiopia. Natl Guidel Fam Plan Serv Ethiop 2019. 2019;(July):1–65.

29. Tao J, Ling J, Shan T, Chi M. Effectiveness of a theory-based postpartum sexual health education program on women ’ s contraceptive use : a randomized controlled trial ☆. Contraception [Internet]. 2011;84(1):48–56.

30. Lori JR, Chuey M, Munro-kramer ML, Ofosu-darkwah H, Adanu RMK. Increasing postpartum family planning uptake through group antenatal care : a longitudinal prospective cohort design. 2018;1–8.

31. Shiferaw S, Spigt M, Tekie M, Abdullah M. The Effects of a Locally Developed mHealth Intervention on Delivery and Postnatal Care Utilization ; A Prospective Controlled Evaluation among Health Centres in Ethiopia. 2016;1–14.

32. Ntambuto RM. No Title.

33. Bekele D, Surur F, Nigatu B, Teklu A, Getinet T, Kassa M, et al. Knowledge and Attitude Towards Family Planning Among Women of Reproductive Age in Emerging Regions of Ethiopia. 2020;

34. Shaaban OM, Saber T, Youness E, Farouk M, Abbas M. Effect of a mobile phone-assisted postpartum family planning service on the use of long-acting reversible contraception : a randomised controlled trial. Eur J Contracept Reprod Heal Care [Internet]. 2020;25(4):264–8.

35. Shiferaw S, Workneh A, Yirgu R, Dinant G, Spigt M. Designing mHealth for maternity services in primary health facilities in a low-income setting – lessons from a partially successful implementation. 2018;9:1–14.

36. Dangal G, Hospital KM, Kutty R. Construction and Validation of a Women ’ s Autonomy Measurement Scale with Reference to Utilization of Maternal Health Care Services in Nepal. 2015;(May 2017).

37. Chakraborty NM, Fry K, Behl R, Longfield K. Simplified asset indices to measure wealth and equity in health programs: A reliability and validity analysis using survey data from 16 countries. Glob Heal Sci Pract. 2016;4(1):141–54.

38. Helwig NE, Hong S, Hsiao-wecksler ET. DHS toool to scoio economic assesment, 2019

**Annexes**

Annex I: **Table 1:** Mobile health (mHealth) intervention schedule and package/Protocol on effectiveness of mobile health intervention to improve early postpartum modern contraception method adoption at Dessie and Kombolcha, north east Ethiopia.

| **Sr. No** | **Week** | **Intervention package/Protocol** |
| --- | --- | --- |
| 1 | 30^th^ weeks of gestation | “Good health to you. To improve the health of the mother and the child the mother should birth space of 2-3 years after child birth. “Dear sir, mother do the following accordingly to improve the health of the mother and neonate Plan to take birth control method to take early after child birth to prevent short birth interval. If you have any question/need of clarification related the message you can miscall/call with this mobile number. |
| 2 | 32th weeks of gestation | “Good health to you. “Dear sir, mother Plan to take birth control method to take early after child birth within 45 days after delivery to prevent short birth interval. After birth contact the health care provider to choose contraceptive methods to take early and prevent unwanted pregnancy and narrow birth interval. If you have any question/need of clarification related the message you can miscall/call with this mobile number. |
| 3 | 34^th^ weeks of gestation | “Hello good health to you. “Dear Sir, there is a need of birth spacing minimum of 2-3 years. Sometimes a woman can be pregnant starting from 45 days after child birth even without showing menstruation if she has sexual practice. To prevent this decide to take early postpartum family planning after child birth any time starting from immediately after birth to 45 days after”. If you have any question/need of clarification related the message you can miscall/call with this mobile number. |
| 4 | 36^th^ weeks of gestation | “Hello good afternoon. There is a need of birth spacing minimum of 2-3 years. Sometimes a woman can be pregnant starting from 45 days after child birth even without showing menstruation if she has sexual practice. To prevent this decide to take early postpartum family planning after child birth any time starting from immediately after birth to 45 days after”. If you have any question/need of clarification related the message you can miscall/call with this mobile number |
| 5 | 38^th^ weeks of gestation | “Good health to you. The time to delivery is reaching and does the following. Decide to give birth at health facility, Prepare emergency transport, and Prepare social support to health facility. Dear sir mother, Based on world health organization recommendation a woman should have birth space of 2-3 years to the health of the mother and thichild. To prevent narrow birth interval and unwanted pregnancy discuss among your husband and decide to take early postpartum family planning after any time starting from immediately after birth to 45 day”. If you have any question/need of clarification related the message you can miscall/cal. |
| 6 | 40^th^ weeks of gestation | Dear sir mother, To prevent narrow birth interval and unwanted pregnancy decide to take early postpartum family planning after any time starting from immediately after birth to 45 day”. If you have any question/need of clarification related the message you can miscall/call with this mobile number. |
| 7 | 2^nd^ week postpartum | Dear sir mother, Based on world health organization recommendation a woman should have birth space of 2-3 years to the health of the mother and thichild. To prevent narrow birth interval and unwanted pregnancy discuss among your husband and decide to take early postpartum family planning after any time starting from immediately after birth to 45 day”. If you have any question/need of clarification related the message you can miscall/cal. |
| 8 | 4^th^ week postpartum | Dear sir mother, Based on world health organization recommendation a woman should have birth space of 2-3 years to the health of the mother and thichild. To prevent narrow birth interval and unwanted pregnancy discuss among your husband and decide to take early postpartum family planning after any time starting from immediately after birth to 45 day”. If you have any question/need of clarification related the message you can miscall/cal. |
