# Supplementary material for: Effectiveness of an Interactive Mobile Health Intervention (IMHI) to enhance the adoption of modern contraceptive methods during the early postpartum period among women in Northeast Ethiopia: A cluster Randomized Controlled Trial (RCT)
Source: PLoS One. 2024 Nov 14;19(11):e0310124. doi: 10.1371/journal.pone.0310124 (PMC11563424; doi:10.1371/journal.pone.0310124)
Supplement: S3 File — (DOCX) [file pone.0310124.s004.docx]

**Supporting information**

**Supporting information 1:** Mobile health (mHealth) intervention schedule and Sending Message Service (SMS) package on the effectiveness of mobile health intervention to improve early postpartum modern contraception method adoption at Dessie and Kombolcha cities, northeast Ethiopia.

| **Sr. No** | **Week** | **Intervention package/Protocol** |
| --- | --- | --- |
| 1 | 30^th^ weeks of gestation | "Good health to you. The time to delivery is reaching and does the following. Decide to give birth at a health facility, Prepare emergency transport, and Prepare social support for the health facility. Dear Sir Mother, Based on the World Health Organization recommendation a woman should have a birth space of 2-3 years for the health of the mother and the child. To prevent narrow birth interval and unwanted pregnancy discuss among your husband and decide to take early postpartum family planning after any time starting from immediately after birth to 45 days". If you have any questions/need clarification related to the message you can miscall/call. |
| 2 | 32th weeks of gestation | "Good health to you. “Dear sir, the mother plans to take the birth control method early after childbirth within 45 days after delivery to prevent short birth intervals. After birth contact the health care provider to choose contraceptive methods to take early and prevent unwanted pregnancy and narrow birth interval. If you have any questions/need clarification related to the message you can miscall/call with this mobile number. |
| 3 | 34^th^ weeks of gestation | "Hello, good health to you. “Dear Sir, there is a need for birth spacing minimum of 2-3 years. Sometimes a woman can be pregnant starting from 45 days after childbirth even without showing menstruation if she has sexual practice. To prevent this decide to take early postpartum family planning after childbirth any time starting from immediately after birth to 45 days after". If you have any questions/need clarification related to the message you can miscall/call with this mobile number. |
| 4 | 36^th^ weeks of gestation | "Hello, good afternoon. There is a need for birth spacing a minimum of 2-3 years. Sometimes a woman can be pregnant starting from 45 days after childbirth even without showing menstruation if she has sexual practice. To prevent this decide to take early postpartum family planning after childbirth any time starting from immediately after birth to 45 days after". If you have any questions/need clarification related to the message you can miscall/call this mobile number |
| 5 | 38^th^ weeks of gestation | “Good health to you. To improve the health of the mother and the child the mother should birth space of 2-3 years after childbirth. “Dear sir, mother do the following accordingly to improve the health of the mother and neonate Plan to take birth control method early after childbirth to prevent short birth intervals. If you have any questions/need clarification related to the message you can miscall/call with this mobile number. |
| 6 | 40^th^ weeks of gestation | Dear sir mother, To prevent narrow birth interval and unwanted pregnancy decide to take early postpartum family planning at any time starting from immediately after birth to 45 days". If you have any questions/need clarification related to the message you can miscall/call with this mobile number. |
| 7 | 2^nd^ week postpartum | Dear Sir Mother, Based on the World Health Organization recommendation a woman should have a birth space of 2-3 years for the health of the mother and the child. To prevent narrow birth interval and unwanted pregnancy discuss among your husband and decide to take early postpartum family planning after any time starting from immediately after birth to 45 days". If you have any questions/need clarification related to the message you can miscall/call. |
| 8 | 4^th^ week postpartum | Dear Sir Mother, Based on the World Health Organization recommendation a woman should have a birth space of 2-3 years for the health of the mother and the child. To prevent narrow birth interval and unwanted pregnancy discuss among your husband and decide to take early postpartum family planning after any time starting from immediately after birth to 45 days". If you have any questions/need clarification related to the message you can miscall/call. |
